# High unawareness of kidney dysfunction in European older adults and the importance of early detection through comorbidities

**DOI:** 10.1371/journal.pone.0333578

**Published:** 2025-10-14

**Authors:** Hannah Marie Horton, Martina Börsch-Supan

**Affiliations:** 1 Technical University of Munich, Munich, Bavaria, Germany; 2 Department of Health Econometrics, Munich Research Institute for the Economics of Aging and SHARE Analyses, Munich, Bavaria, Germany; 3 SHARE-ERIC Biomarker Project, Munich, Bavaria, Germany; Istanbul University-Cerrahpasa, Cerrahpasa Medical Faculty, TÜRKIYE

## Abstract

**Introduction:**

Chronic kidney disease (CKD) continues to go undiagnosed at significant rates in Europe, with few exploring the underlying mechanisms which prompt detection. Considering the causal link of numerous health conditions and CKD, this study investigates how individual and combined comorbidities are associated with the likelihood of CKD detection.

**Materials and methods:**

In a large population study (n = 22,386) of older adults (50+) among 11 European countries and Israel, we calculate the prevalence of undiagnosed CKD using serum-equivalent cystatin C values, derived from dried blood spots. Logistic regressions are estimated to predict factors related to CKD diagnosis among the population and among those with diagnosed vs. undiagnosed CKD.

**Results:**

Unawareness of CKD among older adults is estimated at 11% in the population and ~85% among CKD cases, with country heterogeneity. Common conditions which should prompt screening, such as hypertension and diabetes, do not increase the likelihood of CKD diagnosis among those with reported and measured CKD. Instead, conditions which are demanding in terms of pain and continuous care, such as cancer and arthritis, are associated with an increased likelihood of CKD detection. However, even in the case of four or more comorbidities, the likelihood of CKD detection is only 27.3%. Women and older individuals are more likely to remain undiagnosed.

**Conclusions:**

In order to increase the chance of detection, comorbidities need to be better interpreted as early warning signals of CKD.

## Introduction

Chronic kidney disease (CKD) is a global health issue which afflicts nearly 850 million people worldwide [1]. In Europe, it is estimated that around 10–13% of the population is affected by CKD to some degree [2,3], which not only poses a significant burden on individuals, but constitutes a global public health concern as the prevalence continues to rise [3,4]. Furthermore, kidney replacement therapy makes CKD one of the most expensive diseases for health systems, with an estimated EUR 150 billion annually since 2021 in Europe alone [5]. Therefore, in order to delay and prevent the onset of CKD, timely detection through early warning signals is paramount. Alongside high health spending, there is a high human cost of moderate to severe CKD, with a substantial reduction in the quality of life and a substantial increase in mortality risk in afflicted persons [6,7]. This increase is compounded by commonly related comorbidities, with conditions such as diabetes, hypertension, coronary heart disease, stroke, and heart failure being linked to CKD incidence and complication [8–10]. While kidney function declines naturally with age, many of the conditions which further reduce kidney function are also age-related. It has been estimated that approximately 50% of those with later-stage CKD have CVD comorbidity [11], whereas nearly all have multimorbidity [8,10]. While the direction of causality is complex, most CVD comorbidities often precede CKD, as conditions such as high blood pressure or high blood sugar can damage the blood vessels in the kidneys. Though diabetes and cardiovascular comorbidities are the predominant concern, CKD is also associated with some forms of cancer [12], anemia [13], and rheumatoid arthritis [14] among other conditions.

While cures for CKD are still experimental and not currently widespread [15], early detection is key to slow the progression of the disease by managing the precedent comorbidities that are most likely causal for CKD (e.g., CVD or diabetes) and adopting a healthy lifestyle as early as possible [16]. However, an important obstacle to the prevention and treatment of CKD is that a major number of those afflicted remain undiagnosed [17,18]. CKD is divided into six stages of severity (G1, G2, G3A, G3B, G4, and G5). Among those with moderate CKD (G3A), it has been estimated that the rate of undiagnosed cases is over 70% in Europe [17,18], with country heterogeneity. Part of the challenge in detection arises due to the fact that early-stage CKD has few symptoms as damage develops slowly over time. Therefore, CKD is often only caught when the disease has progressed to more severe stages, which delays and limits treatment options. It is therefore crucial to understand the factors which cause some individuals to become diagnosed, while the majority are still left behind. Is having a certain comorbidity or multimorbidity associated with a likelihood of CKD detection?

Presently, very little literature has made country-level comparisons of undiagnosed CKD in Europe, and none have discussed the relationship between comorbidity and diagnosed status. Understanding which countries’ health systems more successfully detect CKD may reveal effective interventions and strategies in targeting CKD and related comorbidities. This study overcomes two prominent challenges faced in the existing literature, country comparability and selection bias. First, we use multinational data, not only with a harmonized questionnaire for self-reported health measures, but more importantly with a centralized lab where respondents’ blood samples signaling decline of kidney function were analyzed under comparable conditions with the same assay. Second, it has been difficult to capture undiagnosed CKD outside of a clinical setting, as individuals who rarely seek medical treatment likely remain undiagnosed, which leads to sampling bias. By reaching individuals in their homes, we are able to capture a more representative sample of older adults who may otherwise go unobserved.

In the present study, we estimate a series of logistic regressions to test the extent to which having a comorbidity or multimorbidity is associated with having diagnosed CKD among the full population, and secondarily among those with reported and measured CKD. Undiagnosed CKD is derived using dried blood spot (DBS) data from the Survey of Health, Ageing, and Retirement in Europe (SHARE) [19] from cystatin C (CysC) blood levels alongside self-reported CKD status. From these measures, we additionally explore the prevalence of diagnosed and undiagnosed CKD in 11 European countries and Israel, to discuss the extent to which comorbidities can explain differences in country prevalence. Gaining an insight into the scope of CKD unawareness and comorbidity overlap, alongside factors that predict CKD detection, will allow health officials to target interventions for at-risk populations.

## Materials and methods

### Sample

Our data come from the blood biomarker collection from SHARE, a large research infrastructure studying the health, social, and economic circumstances of adults aged 50 and older in Europe [20,21]. From January to November 2015, SHARE collected dried blood spot (DBS) data from ~27,000 individuals in 12 participating countries (Belgium (BE), Switzerland (CH), Germany (DE), Denmark (DK), Estonia (EE), Spain (ES), France (FR), Greece (GR), Israel (IL), Italy (IT), Sweden (SE), and Slovenia (SI)) in their place of residence. The inclusion of DBS data during SHARE Wave 6 aimed to provide further objective measures of health in older adults, and to complement self-reported health conditions by the survey respondents, which included a report of CKD diagnosis by a doctor. Details regarding the implementation and collection of DBSs in SHARE have been previously reported [19]. In brief, all panel household respondents in the participating countries were required to give written consent at the time of interview for DBS collection. In some countries (CH, DK, IT, SE), respondents had to be informed about the blood collection with an advance letter. The consent form detailed how the samples would be handled and analyzed in the future, and explained that SHARE would not report lab values back to the respondent. The data collected for this study did not contain any information that could identify individual participants, and authors had no access to identifiable information during or after data collection. Authors accessed the data for research purposes on (29.05.2024). Our study includes data from 22,386 participants from the above-mentioned 12 countries with available CysC information. The mean age of the sample is 68.5 ± 9.39 years, and 56.8% are female.

Kidney function was assessed by the biomarker CysC. Blood values of CysC were measured together with total cholesterol, triglycerides, and c-reactive protein in a shared eluent obtained from a DBS subsample [19,22]. A post-field collection validation experiment with non-SHARE donor samples was performed in 2018 to validate the DBS results for identified fieldwork conditions in the laboratory. Using structured conversion formulae, CysC was corrected for these fieldwork conditions and converted to a serum equivalent value [22,23].

### Calculating the glomerular filtration rate

In order to test kidney performance, clinicians most widely estimate the glomerular filtration rate (GFR) from filtration markers such as serum creatinine (eGFRcr) or CysC (eGFRcys) alongside signs of elevated urine albumin (also referred to as albuminuria). Under the current guidelines, eGFR > 90 mL/min/1.73 m^2^ without albuminuria signifies healthy kidney function (G1), whereas eGFR < 60 mL/min/1.73 m^2^ indicates signs of functional loss (G3A).

Creatinine has been criticized for lacking precision for less severe CKD cases, leading to missed diagnosis in the early stages [24], whereas CysC has been found to detect more mild changes in kidney function, and is less subject to the effects of age, sex, and race [25–27]. Hence, it may be advantageous to use CysC over creatinine especially in older adults, allowing a more precise measurement for those with age-related loss of muscle mass or chronic conditions [26,28]. While there are many advantages of cystatin C, the use of both filtration markers in tandem has been shown to give the most precise GFR determination, though marker-specific equations did not return biased estimates [25,28].

We take advantage of the findings from Inker et al. (2012) [25], who estimate the eGFR using CysC and creatinine-based equations. As creatinine could not be obtained from SHARE DBS, we convert CysC values to the glomerular filtration rate (eGFRcys) using the following equations:


$$CysC\leq \text{0}.\text{8}mg/L:\text{1}\text{3}\text{3}\times {\left(\frac{CysC}{\text{0}.\text{8}}\right)}^{-\text{0}.\text{4}\text{9}\text{9}}\times {\text{0}.\text{9}\text{9}\text{6}}^{Age}[\times \text{0}.\text{9}\text{3}\text{2}iffemale$$ (1)

$$CysC>\text{0}.\text{8}mg/L:\text{1}\text{3}\text{3}\times {\left(\frac{CysC}{\text{0}.\text{8}}\right)}^{-\text{1}.\text{3}\text{2}\text{8}}\times {\text{0}.\text{9}\text{9}\text{6}}^{Age}[\times \text{0}.\text{9}\text{3}\text{2}iffemale$$ (2)

From this conversion, we estimate that 11.5% fall into G1, 76.6% into G2, 10% into G3A, 1.7% into G3B, and 0.2% into G4 among the full population. As our sample population is older (50+), it is likely that the majority fall into the G2 category due to normal kidney aging [29], alongside comorbidity-driven kidney damage.

### Defining diagnosed vs. undiagnosed kidney dysfunction

**Fig 1:**
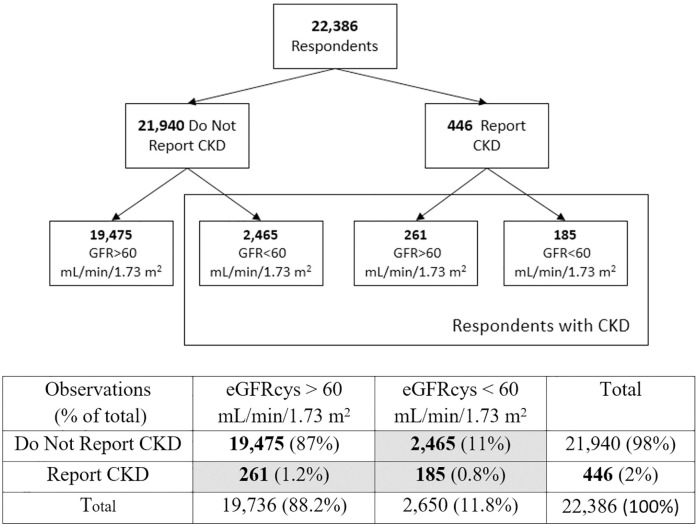
CKD groups defined by self-report and eGFR levels. Note: eGFRcys levels were constructed via CysC-only equations from Inker et al. 2012. Those we classify as “having CKD” (i.e., either through self-report or GFR level) are shown in blue (n = 2911). Of the 2650 cases of moderate to severe kidney impairment (eGFRcys < 60 mL/min/1.73 m^2^), 84.4% fell into stage G3A, 14.1% in G3B, and 1.4% in G4. We did not find any cases in G5. Given the hardship of severe CKD, it is unlikely these individuals would participate in a household survey.

In order to define undiagnosed moderate to severe CKD in the sample, we use whether the respondent reported CKD diagnosis and a fixed cutoff of eGFRcys < 60 mL/min/1.73 m^2^, following the KDIGO (Kidney Disease: Improving Global Outcomes) classification [30]. Fig 1 presents how the sample is organized. Of the 22,386 respondents, 446 (2%) report CKD diagnosis, with 58.5% (n = 261) having eGFRcys levels corresponding to stages G1 and G2, and 41.5% (n = 185) showing signs of greater functional loss (G3A and below). We classify 11% (n = 2465) as undiagnosed CKD, as these cases do not report CKD diagnosis yet have an eGFRcys < 60 mL/min/1.73 m^2^ based on our estimates. Therefore, the overall prevalence of CKD in our sample of older adults (n = 2911) is around 13%, with 84.7% of those cases classified as being undiagnosed. The prevalence of moderate to severe CKD in our sample is 11.8% (n = 2650).

### Statistical analysis

Descriptive statistics for continuous variables include mean and standard deviation, whereas frequencies and percentages characterize categorical and dichotomous variables. Comparative analyses between groups were conducted using Student’s t-test or chi-square tests, depending on variable type and distribution. Following the CKD group organization from Fig 1, in the present study we estimate a series of logistic regressions to develop six prediction models for three diagnosis outcomes in order to identify factors associated with becoming diagnosed with CKD among the full population as well as among those with diagnosed and measured CKD. In Models (1, 2), P(Diagnosed) refers to the probability of reporting CKD (n = 446) among the full population (n = 22,386). In Models (3, 4), P(Diagnosed | CKD) refers to the probability of reporting CKD (n = 446) among those we categorize as having CKD (n = 2911), effectively comparing the difference between diagnosed and undiagnosed CKD groups. Lastly, in Models (5,6), P(Diagnosed | GFR < 60 mL/min/1.73 m^2^) refers to those who report CKD (n = 185) among those who we categorize in the moderate to severe kidney impairment category (n = 2650). As our data does not include a measure of the urine albumin to creatinine ratio (UACR), our measure of undiagnosed CKD cases may be slightly underestimated, and therefore less healthy than their diagnosed counterparts by construction. Therefore, we exclude cases of those with GFR > 60 mL/min/1.73 m^2^ as a further sensitivity analysis, in order to only capture cases which fall below G3A. While Models (1), (3), (5) use individual comorbidities as covariates, Models (2), (4), (6) employ a cumulative risk index for the number of comorbidities a respondent reports between diabetes, hypertension, heart attack, stroke, arthritis, and cancer. Results are expressed in terms of odds ratios (OR) with 95% confidence intervals (CI) shown in parentheses. To assess model performance, we conduct k-fold cross validation (k = 5) with stratification by CKD status to maintain class distribution across folds. Final performance metrics are pooled across folds using mean AUC with 95% confidence intervals derived from standard errors of the k estimates, using Rubin’s rules to adjust for sampling weights.

Control covariate selection was derived from a combination of known theoretical linkages of diagnosis guided by established CKD risk factors, robustness against omitted variable bias via sensitivity analysis comparing nested models, and statistical significance in model specifications.

Our analysis uses the product of the calibrated cross-sectional individual weights available from SHARE Wave 6 and a non-response adjustment factor to account for non-randomness of DBS participation. For more information on our weighting strategy, see [19,31]. Weights are applied as probability weights in each model. Other health covariates (BMI, smoking, physical inactivity, and depression) are pulled from a generated dataset that imputed missing values using the hot deck method [31]. Furthermore, demographic controls (age, gender, education, country) are included in each model.

All analysis was conducted in STATA 14.2

### Sample characteristics

The descriptive statistics are presented in Table 1. On average, those with undiagnosed CKD (52.6 mL/min/1.73 m^2^) have lower eGFR levels than their diagnosed counterparts (61.8 mL/min/1.73 m^2^ (p < 0.001)). However, as the diagnosed cases include those in earlier stages of CKD, the group’s average eGFR is higher by definition. When restricting both measures to eGFR < 60 mL/min/1.73 m^2^, those with diagnosed CKD have an average eGFR level of 44.5 mL/min/1.73 m^2^ (p < 0.001). Those with diagnosed CKD tend to have higher rates of common comorbidities than their undiagnosed counterparts, despite being younger on average, 72.4 years vs. 78.9 years (p < 0.001). Among diagnosed individuals, 90.1% have at least one comorbidity, compared to 83.9% of undiagnosed cases. For reference, among the total population, 36.1% have no comorbidities. In addition, undiagnosed cases are more likely to be female, at 66% (SD = .47).

**Table 1:** Baseline characteristics of the study population.

Variable	Total Observations	Total Mean (SD)	Diagnosed CKD (*n = 446*)	Diagnosed CKD (eGFR < 60) (*n = 185*)	Undiagnosed CKD (*n = 2465*)
Cystatin C	*22,386*	**.99 (.13)**	**1.18 (.32)**	**1.47 (.32)**	**1.24 (.14)**
eGFR_cys-SHARE_	*22,386*	**75.01 (12.92)**	**61.8 (17.7)**	**44.49 (10.46)**	**52.6 (6.5)**
Diabetes	*22,386*	**.15 (.36)**	**.32 (.46)**	**.39 (.49)**	**.26 (.44)**
Hypertension	*22,386*	**.42 (.49)**	**.67 (.47)**	**.74 (.44)**	**.61 (.49)**
Heart attack	*22,386*	**.12 (.32)**	**.32 (.47)**	**.39 (.49)**	**.22 (.42)**
Stroke	*22,386*	**.03 (.18)**	**.10 (.30)**	**.12 (.32)**	**.06 (.25)**
Arthritis*	*22,386*	**.26 (.44)**	**.47 (.49)**	**.43 (.49)**	**.34 (.47)**
Cancer	*22,386*	**.04 (.20)**	**.12 (.33)**	**.15 (.34)**	**.06 (.23)**
EURO-D depression	*22,386*	**.26 (.43)**	**.49 (.50)**	**.50 (.50)**	**.37 (.48)**
Number of Comorbidities					
None	*8,090 (36.1%)*		*44 (9.9%)*	*13 (7%)*	*397 (16.1%)*
One	*7,985 (35.7%)*		*123(27.6%)*	*44 (23.8%)*	*867 (35.2%)*
Two	*4,315 (19.3%)*		*123(27.6%)*	*51 (27.6%)*	*726(29.4%)*
Three	*1,575 (7.0%)*		*105(23.5%)*	*49 (26.5%)*	*361 (14.6%)*
Four or more	*421 (1.9%)*		*51 (11.4%)*	*28 (15.1%)*	*114 (4.6%)*
BMI	*22,386*	**27.0 (4.67)**	**28.6 (5.9)**	**28.34 (6.23)**	**27.8 (5.2)**
Ever smoke?	*22,386*	**.47 (.50)**	**.43 (.49)**	**.43 (.50)**	**.39 (.49)**
Alcohol in last 7 days?	*22,386*	**.57 (.49)**	**.39 (.49)**	**.36 (.48)**	**.43 (.49)**
Physical inactivity	*22,386*	**.09 (.28)**	**.26 (.44)**	**.39 (.49)**	**.22 (.41)**
Education					
Low	*7,576 (33.8%)*		*185 (41.5%)*	*87 (47%)*	*1,241 (50.3%)*
Medium	*8,947 (40%)*		*167 (37.4%)*	*59 (31.9%)*	*813 (33%)*
High	*5,863 (26.2%)*		*94 (21.1%)*	*39 (21.1%)*	*411 (16.7%)*
Ability to Make Ends Meet					
With great difficulty	*2,069 (9.2%)*		*73 (16.4%)*	*28 (15.1%)*	*271 (11%)*
With some difficulty	*5,192 (23.2%)*		*131 (29.4%)*	*49 (26.5%)*	*618 (25.1%)*
Fairly easily	*5,774 (25.8%)*		*119 (26.7%)*	*50 (27%)*	*669 (27.1%)*
Easily	*9,351 (41.8%)*		*123 (27.6%)*	*58 (31.4%)*	*907 (36.8%)*
Age in 2015	*22,386*	**68.5 (9.39)**	**72.4 (9.6)**	**76.35 (9.25)**	**78.9 (8.0)**
50-64	*8,423 (37.6%)*		95 (21.3%)	24 (13%)	120 (4.9%)
64-74	*7,794 (34.8%)*		142 (31.8%)	41 (22.2%)	531 (21.5%)
75-84	*4,906 (21.9%)*		164 (36.8%)	87 (47%)	1,177 (47.8%)
85+	*1,263 (5.6%)*		45 (10.1%)	33 (17.8%)	637 (25.8%)
Female	*22,386*	**0.568 (.49)**	**.62 (.48)**	**.54 (.50)**	**.66 (.47)**
Country					
Germany	*2,712 (12.11%)*		*53 (11.9%)*	*21 (11.4%)*	*239 (9.6%)*
Sweden	*2,369 (10.58%)*		*19 (4.3%)*	*16 (8.7%)*	*392 (15.9%)*
Spain	*1,256 (5.61%)*		*26 (5.8%)*	*5 (2.7%)*	*151 (6.1%)*
Italy	*1,599 (7.14%)*		*36 (8.1%)*	*23 (12.4%)*	*160 (6.5%)*
France	*375 (1.68%)*		*7 (1.6%)*	*3 (1.6%)*	*40 (1.6%)*
Denmark	*2,590 (11.57%)*		*30 (6.7%)*	*12 (6.5%)*	*214 (8.7%)*
Greece	*633 (2.83%)*		*9 (2%)*	*6 (3.2%)*	*47 (1.9%)*
Switzerland	*1,791 (8.00%)*		*12 (2.7%)*	*5 (2.7%)*	*254 (10.3%)*
Belgium	*2,929 (13.08%)*		*46 (10.3%)*	*22 (11.9%)*	*296 (12%)*
Israel	*771 (3.44%)*		*34 (7.6%)*	*12 (6.5%)*	*69 (2.8%)*
Slovenia	*1,988 (8.88%)*		*41 (9.2%)*	*18 (9.7%)*	*222 (9%)*
Estonia	*3,373 (15.07%)*		*133(29.8%)*	*42 (22.7%)*	*384 (15.6%)*

*Note: Data are n (%) & mean (SD). Means (SD) shown in bold, observation count n (%) for categorical variables also presented underneath in italics.* eGFR_cys-SHARE_ refers to the glomerular filtration rate estimated by SHARE cystatin C. *Arthritis is a combined measure of both rheumatoid and osteoarthritis from self-report. Education is constructed from the ISCED 1997 classification, where 0–2 (Low), 3–4 (Medium), and 5–6 (High)

Consistent with the literature, hypertension and diabetes are quite common among CKD cases, affecting 67% and 31% of diagnosed cases, and 61% and 26% of undiagnosed cases, respectively. In a separate analysis of country-specific prevalence for each related comorbidity, we find a slight regional divide, shown in S1 Table. Southern (GR, IT, ES, IL) and Eastern countries (EE, SI) show higher rates of diabetes and hypertension than their Western neighbors.

## Results

**Fig 2:**
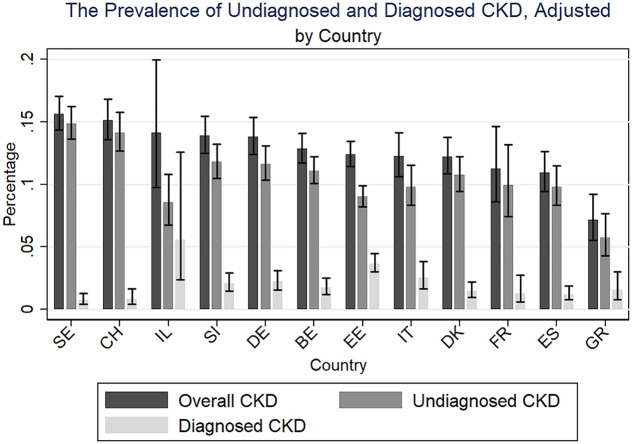
Prevalence of diagnosed and undiagnosed CKD, adjusted. Note: Fig 2 depicts the prevalence of diagnosed and undiagnosed CKD. Estimates were obtained from a logistic regression adjusted for gender, age, and education, and employed DBS-specific weights. Predictive margins by country are presented. Fig 2 is sorted by adjusted overall CKD prevalence. Estimates from France and Greece should be met with greater scrutiny due to lower sample size. Sweden (SE), Switzerland (CH), Israel (IL), Slovenia (SI), Germany (DE), Belgium (BE), Estonia (EE), Italy (IT), Denmark (DK), France (FR), Spain (ES), Greece (GR).

**Fig 3:**
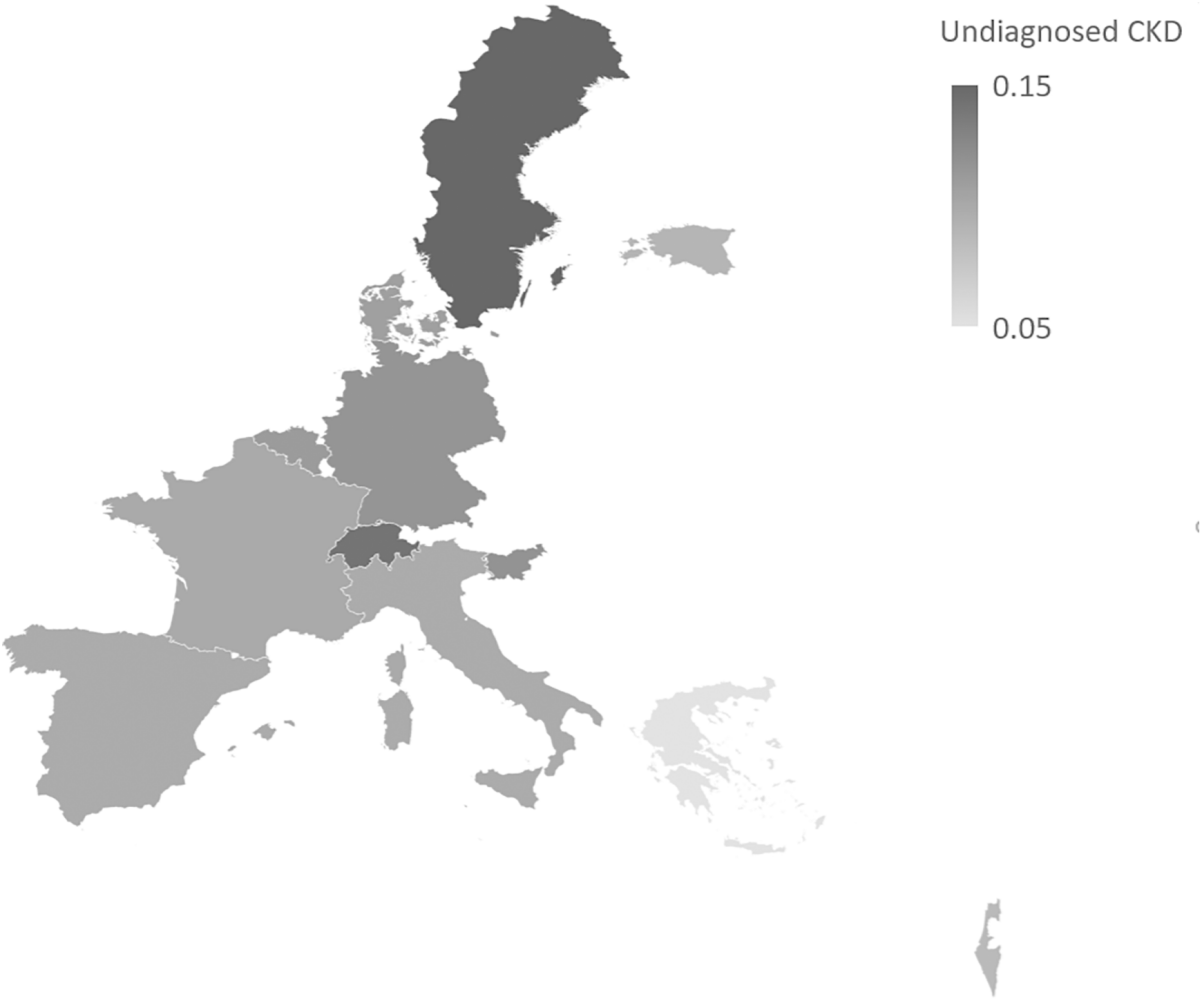
Prevalence estimates of undiagnosed CKD, adjusted. Note: Fig 3 depicts undiagnosed CKD estimates in Europe and Israel, which were obtained from a logistic regression adjusted for gender, age, and education, and employed DBS-specific weights. Predictive margins by country are presented, precise values found in Fig 2.

Using the converted eGFRcys values, we estimate the adjusted prevalence of total, diagnosed, and undiagnosed CKD by country, shown in Fig 2. In our sample of older European adults, the overall adjusted prevalence of CKD ranges from 10–15% between countries. These estimates are highest in Sweden (15.6%) and Switzerland (15.1%), which are driven by the highest rates of undiagnosed CKD at 14.8% and 14.1%, respectively. The lowest overall prevalence estimates of total CKD are from France (11.3%), Spain (10.9%), and Greece (7.1%). While the estimates from France and Greece should be met with scrutiny due to small sample size, findings are consistent with the literature [32,33]. The prevalence of undiagnosed CKD by country is presented in Fig 3.

The main results are presented in Table 2. In Model (1), we observe that being diagnosed with diabetes [OR= 1.595, 95% CI = 1.02–2.49, p < 0.040], heart attack [OR= 1.741, 95% CI = 1.03–2.93, p < 0.037], arthritis [OR= 2.22, 95% CI = 1.45–3.40, p < 0.000], and cancer [OR= 4.07, 95% CI = 2.36–7.03, p < 0.000] each increase the likelihood one is also diagnosed with CKD among the full population. Similarly, in Model (2), the likelihood of detection is increasing in the number of comorbidities one reports, with those with four or more comorbidities [OR= 14.58, 95% CI = 5.29–40.21, p < 0.000] being more likely to be diagnosed than those with no comorbidities. Among both full population specifications, scoring high (>4) on the EURO-D scale for symptoms of depression [OR = 1.86, 95% CI = 1.16–3.00, p < 0.009] is associated with an increased chance of being diagnosed with CKD. Similarly, those who report that they hardly ever/never engage in physical activity [OR = 1.64, 95% CI = 1.04–2.58, p < 0.032] are more likely to be diagnosed. Among the full population, we do not observe any evidence that demographics play a role in the likelihood of CKD detection.

**Table 2:** Likelihood of comorbidity on CKD detection.

	Model (1)	Model (2)	Model (3)	Model (4)	Model (5)	Model (6)
VARIABLES	P(Diag)	P(Diag)	P(Diag|CKD)	P(Diag|CKD)	P(Diag | GFR < 60)	P(Diag | GFR < 60)
Diabetes	1.595** (1.02 - 2.49)		1.161 (0.67 - 2.01)		1.716 (0.82 - 3.60)	
Hypertension	1.389 (0.84 - 2.30)		0.722 (0.42 - 1.23)		0.770 (0.39 - 1.51)	
Heart attack	1.741** (1.03 - 2.93)		1.267 (0.68 - 2.35)		2.240** (1.09 - 4.58)	
Stroke	1.967 (0.96 - 4.02)		1.460 (0.71–3.00)		1.652 (0.70 - 3.88)	
Arthritis*	2.221*** (1.45 - 3.40)		2.981*** (1.81 - 4.92)		2.039** (1.04 - 3.99)	
Cancer	4.075*** (2.36 - 7.03)		4.491*** (2.20 - 9.16)		5.957*** (2.65 - 13.38)	
Number of comorbidities						
One		3.137*** (1.51 - 6.52)		2.429 (0.99 - 5.95)		7.420*** (1.86 - 29.60)
Two		3.439*** (1.66 - 7.12)		1.786 (0.74 - 4.28)		4.723** (1.07 - 20.87)
Three		9.245*** (4.33 - 19.72)		4.749*** (1.88 - 12.02)		16.34*** (3.85 - 69.25)
Four or more		14.58*** (5.29 - 40.21)		5.970*** (2.02 - 17.65)		27.28*** (5.55 - 134.20)
Euro-D	1.867*** (1.16–3.00)	1.918*** (1.20 - 3.06)	1.368 (0.82 - 2.27)	1.466 (0.88 - 2.43)	1.619 (0.77 - 3.39)	1.787 (0.85 - 3.78)
BMI	1.004 (0.95 - 1.06)	1.001 (0.95 - 1.06)	0.953 (0.90 - 1.00)	0.945** (0.89 - 1.00)	0.952 (0.88 - 1.03)	0.944 (0.87 - 1.03)
Ever smoke?	0.969 (0.61 - 1.55)	0.948 (0.60 - 1.51)	0.817 (0.47 - 1.41)	0.864 (0.50 - 1.48)	1.135 (0.52 - 2.47)	1.249 (0.57 - 2.72)
Physical inactivity	1.642** (1.04 - 2.58)	1.665** (1.06 - 2.60)	1.599 (0.95 - 2.69)	1.703** (1.03 - 2.82)	1.542 (0.82 - 2.89)	1.654 (0.90 - 3.02)
Alcohol in last 7 days?	0.816 (0.50 - 1.34)	0.820 (0.50 - 1.36)	1.067 (0.62 - 1.82)	1.188 (0.71 - 1.99)	1.463 (0.71 - 3.02)	1.504 (0.74 - 3.05)
Ability to make ends meet						
With some difficulty	0.820 (0.44 - 1.54)	0.821 (0.43 - 1.56)	0.715 (0.34 - 1.49)	0.815 (0.41 - 1.66)	0.492 (0.20 - 1.18)	0.588 (0.25 - 1.36)
Fairly easily	0.960 (0.46 - 1.99)	0.929 (0.45 - 1.93)	0.926 (0.42 - 2.04)	0.960 (0.44 - 2.09)	0.604 (0.21 - 1.73)	0.617 (0.22 - 1.69)
Easily	1.090 (0.56 - 2.13)	1.046 (0.53 - 2.07)	1.227 (0.57 - 2.63)	1.264 (0.61 - 2.63)	0.605 (0.23 - 1.55)	0.594 (0.23 - 1.52)
Education						
Medium educ	0.674 (0.40 - 1.15)	0.701 (0.42 - 1.18)	0.766 (0.44 - 1.34)	0.715 (0.40 - 1.27)	0.744 (0.35 - 1.60)	0.680 (0.32 - 1.43)
High educ	0.873 (0.42 - 1.84)	0.932 (0.45 - 1.94)	0.653 (0.31 - 1.35)	0.640 (0.32 - 1.27)	1.143 (0.44 - 2.95)	1.166 (0.51 - 2.66)
Age in 2015	1.020 (1.00 - 1.04)	1.020 (0.99 - 1.04)	0.897*** (0.87 - 0.92)	0.906*** (0.88 - 0.93)	0.930*** (0.89 - 0.97)	0.936*** (0.89 - 0.98)
Female	0.951 (0.57 - 1.59)	0.969 (0.58 - 1.61)	0.513** (0.30 - 0.89)	0.599 (0.35 - 1.03)	0.544 (0.27 - 1.11)	0.561 (0.26 - 1.21)
Country controls	X	X	X	X	X	X
Pooled AUC	0.746 (0.67 - 0.82)	0.749 (0.68 - 0.82)	0.754 (0.67 - 0.84)	0.749 (0.67 - 0.83)	0.671 (0.52 - 0.82)	0.673 (0.53 - 0.81)
Observations	22,386	22,386	2,911	2,911	2,650	2,650

Note: Odds ratios presented with 95% in parentheses (*** p < 0.01, ** p < 0.05). Models (1), (2) predict the probability of CKD diagnosis among the full sample. Models (3), (4) predict the probability of CKD diagnosis among those with reported and measured CKD. Models (5), (6) predict the probability of CKD diagnosis among those with reported and measured CKD, with eGFRcys levels below 60 mL/min/1.73 m^2^*. Arthritis is a combined measure of both rheumatoid and osteoarthritis from self-report. Country controls included in each model.*

Turning to Models (3) and (4), which only include those with diagnosed and undiagnosed CKD, a similar pattern emerges. However, in Model (3) there is no evidence of a significant difference between diagnosed and undiagnosed groups when it comes to the most common cardiovascular comorbidities, diabetes, and hypertension. Instead, only arthritis [OR= 2.98, 95% CI = 1.81–4.92, p < 0.000] and cancer [OR= 4.49, 95% CI = 2.20–9.16, p < 0.000] show an increased likelihood of detection among those with CKD. Similar to the full model specification, we observe that the likelihood of CKD detection is increasing in the number of comorbidities one has [OR= 5.970, 95% CI = 2.02–17.65, p < 0.001].

As a further sensitivity analysis, we restrict the CKD group to include only those with GFR < 60 mL/min/1.73 m^2^, as only including cases under G3A may provide a more suitable comparison between diagnosed and undiagnosed groups. Similar to the previous specification, Model (5) shows that arthritis [OR= 2.04, 95% CI = 1.04–3.99, p < 0.037] and cancer [OR= 5.96, 95% CI = 2.65–13.38, p < 0.000] increase the likelihood of CKD diagnosis, as well as heart attack [OR= 2.24, 95% CI = 1.09–4.58, p < 0.027]. In this restricted sample, Model (6) shows the likelihood of diagnosis increases tremendously in the number of comorbidities one has.

For further presentation of age-related subanalyses, please refer to supplementary S2–S8 Tables. In brief, certain conditions, like hypertension, may prompt CKD detection in earlier ages (50–64), but not in older groups. Cancer, however, remains a consistent predictor of CKD detection in every age group. We do not find evidence of an interaction effect of age group by number of comorbidities. Instead, the likelihood of CKD detection is increasing in the number of comorbidities, independent of age group.

**Fig 4:**
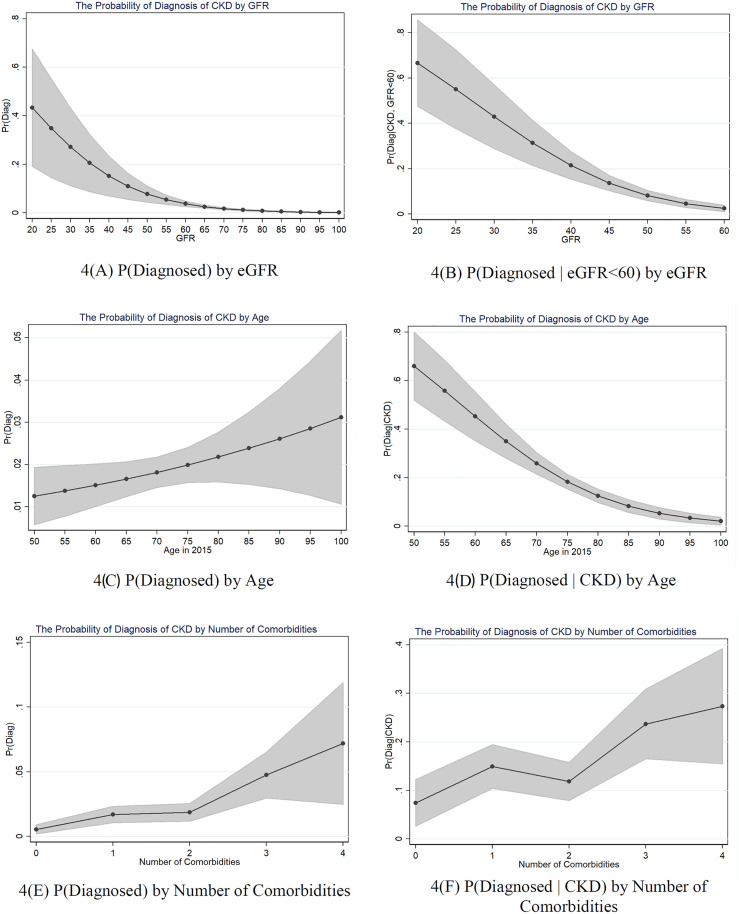
Margins plots for eGFRcys, age, and number of comorbidities. Note: Margins are estimated using models including the number of comorbidities, health, demographic, and country controls. Models are weighted using DBS-specific probability weights.

In Fig 4 we present the marginal effects for GFR level, age and number of comorbidities on the likelihood of diagnosis, with the other covariates held at their means. On the left-hand side, we present the margins using Model (2), the probability of diagnosis among the full population. On the right, we present the margins using Models (4) and (6), the likelihood of diagnosis among those with reported and measured CKD. Figs 4A and 4B show that the likelihood of diagnosis is decreasing in eGFRcys level. However, even for those with very low GFR levels, CKD remains rarely detected. Among those with CKD, those with GFR = 45 (G3B) have a 13.6% chance of reporting CKD, compared to 31.4% at GFR=30 (G4). Turning to Figs 4C and 4D, while CKD diagnosis is increasing in age among the full population, among those with CKD diagnosis becomes rarer with age. Older individuals are much more likely to remain undiagnosed, with those >75 having a less than 18.3% chance of detection. Figs 4E and 4F show that CKD detection is increasing in the number of comorbidities that one has. Among those with CKD, those without any comorbidities have a 7.4% chance of detection, whereas those with 4 or more comorbidities have a 27.3% chance of detection.

## Discussion

In the present study, we examine the relationship between certain comorbidities, the number of comorbidities, as well as demographic and lifestyle factors that influence the likelihood of becoming diagnosed with CKD, among both the full population as well as among those with reported and measured CKD. Despite substantial recent efforts to raise awareness among nephrologists, general physicians and the public, patient awareness of CKD remains low. This trend continues years after our 2015 data collection. One possible explanation is that related comorbidities are not taken seriously enough as early warning signs for CKD [34,35].

Our results indicate that the prevalence of undiagnosed CKD among older adults is very high in Europe (11%), which is in line with estimations from previous literature [2,3]. We observe country variation in CKD prevalence, especially in the undiagnosed category, with Sweden (14.8%) and Switzerland (14.1%) revealing a large population in the GFR category G3A. For some, this finding may seem paradoxical, as both are high income countries with high healthcare coverage and spending. However, this finding is consistent within the literature, which reports that the prevalence of moderate to severe CKD in these countries is relatively common, despite low recognition rates [2,36–38]. While we do not observe a strong regional divide when it comes to overall CKD prevalence, in our sample Eastern countries (EE) and Southern countries (IT, GR, IL) tend to be better at detecting CKD. As will become evident, the potential factors behind these detection differences are complex and largely speculative, which we explore below.

Some have argued that diagnosing CKD may be less incentivized in countries with public health systems, as an official diagnosis has fewer rewards in a government-funded healthcare system than in an insurance or claims system [17,39]. Yet Switzerland, Estonia, Greece, and Israel have insurance-based systems, while Sweden and Italy have publicly funded systems.

Unlike some other countries sampled, both Sweden and Switzerland have no national screening program for CKD. Instead, the healthcare systems are largely decentralized, with regional authorities being responsible for healthcare provision. While Sweden published guidelines for annual CKD screening for high-risk individuals in 2021, a recent study showed that annual screening rates were still notably low even in those with diabetes and hypertension [40]. As of 2025, Switzerland’s National Strategy for the Prevention of Non-Communicable Diseases does not mention CKD, and CKD is not officially considered in any health policy strategy, leading experts to fear that Switzerland is lagging behind [41]. However, among the better-detection countries, none have national CKD screening programs in place either.

Despite the lack of national screening programs, there are country differences in how health officials handle CKD, and how actively they pursue prevention strategies. In 2009, the Italian Society of Clinical Biochemistry and Laboratory Medicine (SIBioC) released official recommendations for CKD, later finding encouraging trends in adherence to KDIGO guidelines [42]. Also in Italy, GPs who received specialized training and support by nephrologists led to a substantial increase in the number of ACR and eGFR tests, increasing overall CKD awareness [43].

Differences between countries also arise in part due to how common collection of both eGFR and UACR are in tandem, especially in high-risk groups. In Swedish study of CKD patients, only two-thirds were followed up annually for an eGFR test and only a quarter for UACR [40]. In comparison, 80% of early-stage CKD patients in Estonia were tested at least 1–2 times per year [44]. After low testing rates for albuminuria in the past, since 2020 it has been obligatory to determine UACR in high-risk groups in Estonia [44]. Among those with diabetes, 79% of Israeli patients received both eGFR and UACR annually [45], while just 21% of Swiss diabetics received nephropathy screening in 2017 [46]. Though it is important to note, UACR screening rates remain underutilized, even in countries where overall detection is higher.

Countries also differ in how eGFR is calculated and whether it is routinely included in blood test panels [47]. A European laboratory survey revealed considerable variation in preferred equations, with most labs relying on the 2009 CKD-EPI formula based solely on serum creatinine. In the same study, only 28.5% of labs measured CysC, and only 8.4% used creatinine and CysC in tandem in eGFR determination [47]. Since CysC captures early renal decline and isn’t as subject to muscle mass or age, reliance on creatinine-only testing may lead to missed early-stage CKD cases, especially in older adults.

Southern countries (ES, IT, GR) also tend to have more nephrologists per capita, nearly double that of Sweden and Switzerland [48]. Though the presence of specialists alone does not ensure the effective referral from GP to nephrologist. Even when kidney dysfunction is known, in Denmark only 2.7% were referred to a nephrologist during follow-up [49]. The number of nephrologists may also be related to the demand for care. In our own sample, respondents in Italy, Greece, Israel, and Estonia were more likely to report forgoing medical care due to long waiting times, despite having better CKD detection rates. One may think this finding points to perhaps longer and more comprehensive physician consultations, yet the literature reports the opposite [50]. Instead, the average doctor’s appointment is much longer in Sweden and Switzerland than it is in Estonia, and the aforementioned countries spend much more on health per capita [50].

Another explanation may be due to country differences in lifestyle behaviors (such as in smoking, obesity, or physical activity), which may increase physician awareness and attention towards CKD. This phenomenon has been used to explain higher CKD prevalence in the east [51], known as the East-West gradient for CKD. As lifestyle factors are causal for many cardiovascular comorbidities, they may play a role in CKD screening and detection, explaining higher diagnosis rates in countries with more comorbidities [52]. Shown in S1 Table, hypertension and diabetes, conditions causal for kidney damage, are more common in Southern and Eastern countries than in Sweden and Switzerland. However, we do not find evidence that having one of these conditions increases the likelihood of detection among those with reported and measured CKD in one country more than in others (S2 Table).

Despite widespread guidelines for CKD assessment [30,34], in reality having a CKD diagnosis may instead indicate the severity of the disease progression [53,54]. A European Diabetes/CKD report found that in France (22%), Germany (24%), Italy (13%), and Spain (14%), many did not receive a CKD diagnosis until after more than a year of first experiencing symptoms [55]. Instead, median follow-up in these countries ranged from 2.2 years in France to 3.6 years in Italy [17]. These findings highlight that most countries, despite differences in health systems, access, and demand, generally perform poorly in recognizing early warning signs for CKD.

Our main results illustrate that some conditions like diabetes, heart attack, arthritis, and cancer are positively associated with being diagnosed with CKD among the full population. However, among those with reported and measured CKD, only arthritis (here combined rheumatoid and osteoarthritis) and cancer consistently show a positive relationship with diagnosis. Those with arthritis have chronic inflammation, which has been linked to the development of CKD through a multitude of interrelated pathways, as well as cardiovascular conditions [14,56]. Cancer is a multifaceted disease, with some forms being more directly linked to CKD than others [12]. However, both of the aforementioned conditions are demanding in terms of pain, fatigue, and continuous care, which may increase the likelihood for CKD detection. In contrast, more common conditions for older adults, such as diabetes and hypertension, should prompt consistent CKD screening, but do not always in practice [35]. This result is concerning, as ignoring these prominent yet commonplace comorbidities will lead to functional loss and increase mortality risk [7,8].

Moreover, in each specification we observe that the likelihood of diagnosis is increasing in the number of comorbidities that one has. Despite this, diagnosis remains relatively rare even for respondents with multimorbidity. Among those with CKD, only 27.3% of cases are discovered when a respondent has four or more comorbidities, compared to 7.4% when a respondent has none. In a similar vein, while lower GFR levels are associated with higher chances of detection, the likelihood that someone reports CKD with a measured eGFR=30 (G4) is only 31.4%.

While the severity of CKD and surrounding comorbidities seem to prompt detection, persistent underdiagnosis highlights the need to strengthen both day-to-day clinical practices alongside broader national strategies. Delays in detection may stem from lack of proactive screening protocols in routine care. As CKD is generally asymptomatic in the early stages, physicians may not screen for it until a patient has multiple comorbidities or is truly ill [35]. It may also be the case that physicians are aware of reduced kidney function yet do not explicitly inform patients, instead choosing to target comorbidities, as managing diabetes or hypertension has a direct impact on the kidneys. Yet raising patient awareness could also induce kidney-health specific lifestyle changes to slow the progression of the disease.

Among all model specifications, we find no evidence of an SES gradient in terms of education or financial insecurity when it comes to the likelihood of CKD diagnosis. This result is surprising, as often the relationship between SES and health literacy, healthcare access, and cost are brought forth to explain differences in diagnosis rates. In many studies, low SES is associated with a greater incidence of CKD and higher likelihood of disease progression into end stage renal disease (ESRD) [57]. Furthermore, many of the risk factors associated with CKD such as obesity, multimorbidity, and physical inactivity have known relationships with SES. While education is not an independent predictor of CKD diagnosis, a supplementary analysis of age, gender, and education (S7 Table) reveals that older more educated females are more likely to remain undiagnosed than their male counterparts. In S8 Table, Model 2 shows that among those aged 75–84, medium and high educated males are diagnosed at rates of 24% and 23.2% respectively, compared to the 7.5% and 2.1% of their female counterparts. The gender gap between CKD diagnosis is lessened in younger cohorts. Future researchers may consider a mediating relationship between SES, CVD, and CKD awareness.

Despite our best efforts, this study is not without its limitations. For one, there is the possibility of measurement error in our GFR estimate. As our analysis is based on a singular eGFR measurement, we may inadvertently include some cases of acute kidney injury (AKI). However, since respondents were interviewed at their place of residence and participated in a relatively long survey, we believe the inclusion of AKI cases to be minimal. While CysC has been found to be a consistent and reliable measure of GFR, having an additional measure for GFR, like creatinine, could make our estimates more precise [25]. However, our measure for CysC has been carefully corrected for fieldwork conditions and converted to serum-equivalent values [19]. Moreover, even when using DBS-derived data, our estimates of CKD in Europe closely fit previous projections [2,3]. While our estimate does not meet the criterion for an official diagnosis, at the population level, DBS data seem to generate reliable results, which could help bridge the gap in high CKD unawareness, as DBS is a cheap, minimally-invasive, and accessible alternative to smaller clinical studies.

There is also the potential for measurement error in our estimate of undiagnosed CKD. As we restrict our measure of undiagnosed CKD to those with GFR < 60 mL/min/1.73 m^2^, we lose early-stage cases with albuminuria. A systematic review of 10 studies estimated the prevalence of albuminuria among each GFR stage, finding that moderately increased albuminuria was present in around 1.9–6.5% of G1 cases and 2.2–5.6% of G2 cases [9]. In a related study using American data, researchers using both eGFR and albuminuria to define undiagnosed CKD found that only 9% of measured CKD respondents were aware that they had the disease [58]. Therefore, our measure of undiagnosed cases may be slightly underestimated. However, by restricting diagnosed cases under GFR = 60 mL/min/1.73 m^2^, we create a suitable comparison group for our measure of undiagnosed CKD. Despite this assurance, in an ideal setting we would also have samples of albumin in urine to define CKD groups more precisely.

While the focus of this paper is mainly on moderate CKD, and not necessarily ESRD, we find very few severe cases. According to the CDC (Center for Disease Control and Prevention, USA), it is expected that 2.3% and 1% of CKD cases fall into G4 and G5, respectively [59]. In our sample, we find 1.4% of CKD cases in G4 and no cases in G5, meaning these severe stages are underrepresented. Given the heavy burden of this disease, it is less likely to capture these cases outside a clinical setting.

While we do our best to control for covariates which may explain the differences between diagnosed and undiagnosed CKD cases, there are potentially other confounding factors which we were unable to measure. For example, SHARE does not include information regarding glomerular or genetic conditions which could be related to CKD, such as polycystic kidney disease (PKD) or IgA nephropathy. Other confounding factors, such as a family history of CKD, dietary patterns, environment, and ethnicity, are also unfortunately not available.

Individuals are not always reliable reporters of their own health, with factors like age, education, and the individual number of conditions influencing accuracy [60]. While SHARE respondents have been found to generally be credible self-reporters of their medical history, diseases interfering with daily life have been found to be more reliable than those with inconsistent symptoms [61]. Therefore, it may be that respondents with earlier-stage CKD report less accurately.

Lastly, our analysis does not include the usage of any medications. Of particular interest may be the use of NSAIDs, as they are the primary treatment for arthritis, yet strongly discouraged in those with kidney dysfunction. While there is a variable in SHARE for taking joint pain medication in the past week, we ultimately decided to omit this variable for a couple of reasons. The largest issue is that NSAID use acts as a collider, as it is both influenced by arthritis and CKD diagnosis, and conditioning on it would therefore introduce bias into the model. Secondarily, the variable for joint pain medication does not specify the drug or dosage, making it difficult to isolate NSAID use accurately. Despite this, the extent to which NSAID use is connected to undiagnosed CKD warrants a dedicated analysis, which is beyond the scope of the current study.

Despite these limitations, this study has a valuable addition to the literature as one of the first large multinational European study to use serum-equivalent DBS values of cystatin C to show the prevalence of diagnosed and undiagnosed CKD. Our prevalence estimates are very consistent with previous CKD projections, which show the potential for using DBS to close the CKD awareness gap at the population level. Moreover, we are the first to use multinational data collected concurrently and analyzed with the same assay, reducing bias in country comparison. Although our data is from 2015, recent reports show that very little has changed in our ability to capture CKD cases [34]. We know that CKD screening is important and cost-effective; the sooner an at-risk individual is detected, the more cost-effective such a treatment will be [62]. It is imperative that comorbidities are evaluated as serious warning signals for CKD and that screening increases, especially among older adults.

## Supporting information

S1 Table Descriptive statistics of country-specific comorbidity rates. Note: Unadjusted means in bold, SD in parentheses, n observations per country.
(DOCX)

S2 Table Probability of CKD diagnosis, comorbidity and country interaction. Note: Both models predict the probability of CKD diagnosis among those with reported and measured CKD. Model 1 includes an interaction of diagnosed diabetes and country, and Model 2 includes an interaction of hypertension and country. All controls (health, demographic) and survey weights are included. Odds ratios presented with 95% CI in parentheses (*** p < 0.01, ** p < 0.05).
(DOCX)

S3 Table Probability of CKD diagnosis by age group. Note: Models (1) – (4) predict the probability of CKD diagnosis among the full sample, for each age cohort (50–64), (65–74), (75–84) and (85+). Arthritis is the combined measure of rheumatoid and osteoarthritis from self-report. Country Controls are included in each model. Odds ratios presented with 95% CI in parentheses (*** p < 0.01, ** p < 0.05).
(DOCX)

S4 Table Probability of diagnosis among those with reported and measured CKD, by age group. Note: Models (1) – (4) predict the probability of CKD diagnosis among those with reported and measured CKD, for each age cohort (50–64), (65–74), (75–84) and (85+). Arthritis is the combined measure of rheumatoid and osteoarthritis from self-report. Country Controls are included in each model. Odds ratios presented with 95% CI in parentheses (*** p < 0.01, ** p < 0.05).
(DOCX)

S5 Table Probability of diagnosis among those with reported and measured CKD (GFR < 60), by age group. Note: Models (1) – (4) predict the probability of CKD diagnosis among those with eGFRcys levels below 60 mL/min/1.73 m^2^, for each age cohort (50–64), (65–74), (75–84) and (85+). Country Controls are included in each model. Odds ratios presented with 95% CI in parentheses (*** p < 0.01, ** p < 0.05).
(DOCX)

S6 Table Interaction effects of number of comorbidities and age group. Note: Models (1) predicts the probability of CKD diagnosis among the full sample. Model (2) predicts the probability of CKD diagnosis among those with reported and measured CKD. Models (3) predicts probability of CKD diagnosis among those with reported and measured CKD, with eGFRcys levels below 60 mL/min/1.73 m^2^. Each model includes the categorical by categorical interaction of the number of comorbidities with age groups (50–64), (65–74), (75–84) and (85+), with no comorbidities and age (50–64) as comparison groups. Country and demographic controls are included in each model, replicating Table 3 in the main text. Odds ratios presented with 95% CI in parentheses (*** p < 0.01, ** p < 0.05).
(DOCX)

S7 Table Triple interaction by age group, gender, and education. Note: Models (1) predicts the probability of CKD diagnosis among the full sample. Model (2) predicts the probability of CKD diagnosis among those with reported and measured CKD. Models (3) predicts probability of CKD diagnosis among those with reported and measured CKD, with eGFRcys levels below 60 mL/min/1.73 m^2^. Each model includes the triple interaction of the gender, age group (50–64), (65–74), (75–84) and (85+), and education (low educ =  ISCED 1997 cat 0–2), (medium educ =  ISCED 1997 cat 3,4), (high educ =  ISCED 1997 cat 5,6). Male, age group (50–64), and low education are the baseline categories. Health, demographic, and country controls are included in each model, replicating Table 3 in the main text. Odds ratios presented with 95% CI in parentheses (*** p < 0.01, ** p < 0.05).
(DOCX)

S8 Table Triple interaction by age group, gender, and education, average marginal Effects. Note: Models (1) predicts the probability of CKD diagnosis among the full sample. Model (2) predicts the probability of CKD diagnosis among those with reported and measured CKD. Models (3) predicts probability of CKD diagnosis among those with reported and measured CKD, with eGFRcys levels below 60 mL/min/1.73 m^2^. We present the average marginal effects of Table 8, held at each combination of age group, gender, and education. The margins come from Table 8, which includes the triple interaction of the gender, age group (50–64), (65–74), (75–84) and (85+), and education (low educ =  ISCED 1997 cat 0–2), (medium educ =  ISCED 1997 cat 3,4), (high educ =  ISCED 1997 cat 5,6). Male, age group (50–64), and low education are the baseline categories. Health, demographic, and country controls are included in each model, replicating Table 3 in the main text. (*** p < 0.01, ** p < 0.05).
(DOCX)

S9 Table Probability of CKD diagnosis by diabetes diagnosis group. Note: Odds ratios presented with 95% in parentheses (*** p < 0.01, ** p < 0.05). Undiagnosed Diabetes is defined by having an Hba1c level above 6.5 mg/dL and not reporting a doctor’ diagnosis of diabetes/not taking any medication for diabetes. Model (1) predicts the probability of CKD diagnosis among the full sample. Models (2) predicts the probability of CKD diagnosis among those with reported and measured CKD. Models (3) predicts the probability of CKD diagnosis among those with reported and measured CKD, with eGFRcys levels below 60 mL/min/1.73 m^2^. Health controls include: hypertension, heart attack, stroke, arthritis, depression (euro-d), bmi, physical inactivity, and alcohol consumption in the last 7 days. Demographic controls include: ability to make ends meet, education, age, gender, and country dummies.
(DOCX)
