# Supplementary material for: High unawareness of kidney dysfunction in European older adults and the importance of early detection through comorbidities
Source: PLoS One. 2025 Oct 14;20(10):e0333578. doi: 10.1371/journal.pone.0333578 (PMC12520349; doi:10.1371/journal.pone.0333578)
Supplement: S1 Table — Note: Unadjusted means in bold, SD in parentheses, n observations per country. (DOCX) [file pone.0333578.s001.docx]

| Country | Diagnosed CKD | Diagnosed Diabetes | Diagnosed Hyper-tension | Heart Attack | Stroke | Arthritis | Cancer |
| --- | --- | --- | --- | --- | --- | --- | --- |
| **Germany**  *(n=2,712)* | **.020** (.14) | **.162** (.37) | **.438** (.50) | **.101** (.30) | **.030** (.17) | **.288** (.45) | .**057** (.23) |
| **Sweden** *(n=2,369)* | **.010** (.09) | **.120** (.32) | **.390** (.49) | **.110** (.31) | **.038** (.19) | **.190** (.39) | **.049** (.22) |
| **Spain**  *(n=1,256)* | **.021** (.14) | **.228** (.42) | **.447** (.50) | **.130** (.34) | **.021** (.15) | **.281** (.45) | **.032** (.18) |
| **Italy**  *(n=1,599)* | **.023** (.15) | **.169** (.37) | .**482** (.50) | **.110** (.31) | **.026** (.16) | **.240** (.43) | **.032** (.18) |
| **France** *(n=375)* | **.019** (.14) | **.120** (.33) | .**395** (.49) | **.123** (.33) | **.024** (.15) | **.363** (.48) | **.056** (.23) |
| **Denmark** *(n=2,590)* | **.012** (.11) | **.075** (.26) | **.344** (.48) | **.086** (.28) | **.031** (.17) | **.266** (.44) | **.039** (.19) |
| **Greece** *(n=633)* | **.014** (.12) | **.235** (.42) | **.499** (.50) | **.210** (.41) | **.035** (.18) | **.242** (.43) | **.027** (.16) |
| **Switzer-land** *(n=1,791)* | **.007** (.08) | **.089** (.29) | **.311** (.46) | **.065** (.25) | **.018** (.13) | **.250** (.43) | **.036** (.19) |
| **Belgium** *(n=2,929)* | **.016** (.12) | **.131** (.34) | **.346** (.48) | **.094** (.29) | **.032** (.18) | **.365** (.48) | **.040** (.20) |
| **Israel** *(n=771)* | **.044** (.21) | **.336** (.47) | **.494** (.50) | **.210** (.41) | **.048** (.21) | **.132** (.34) | **.054** (.23) |
| **Slovenia** *(n=1,988)* | **.021** (.14) | **.167** (.37) | **.479** (.50) | **.106** (.31) | **.043** (.20) | **.184** (.39) | **.042** (.20) |
| **Estonia** *(n=3,373)* | **.039** (.19) | **.165** (.37) | **.535** (.50) | **.195** (.40) | **.044** (.20) | **.260** (.44) | **.045** (.21) |
| **Total** *(n=22,386)* | **.020** (.14) | **.150** (.36) | **.425** (.50) | **.120** (.33) | **.033** (.18) | **.259** (.44) | **.043** (.20) |
