# Supplementary material for: High unawareness of kidney dysfunction in European older adults and the importance of early detection through comorbidities
Source: PLoS One. 2025 Oct 14;20(10):e0333578. doi: 10.1371/journal.pone.0333578 (PMC12520349; doi:10.1371/journal.pone.0333578)
Supplement: S2 Table — Note: Both models predict the probability of CKD diagnosis among those with reported and measured CKD. Model 1 includes an interaction of diagnosed diabetes and country, and Model 2 includes an interaction of hypertension and country. All controls (health, demographic) and survey weights are included. Odds ratios presented with 95% CI in parentheses (*** p < 0.01, ** p < 0.05). (DOCX) [file pone.0333578.s002.docx]

|  | **(1)** | **(2)** |
| --- | --- | --- |
| VARIABLES | **P(Diag\|CKD)**  **Diabetes X Country Interaction** | **P(Diag\|CKD)**  **Hypertension X**  **Country Interaction** |
|  |  |  |
| Diagnosed Diabetes | 0.727 (0.300 - 1.762) |  |
| Diagnosed Hypertension |  | 0.808 (0.386 - 1.691) |
|  |  |  |
| Sweden | **0.189***** (0.0807 - 0.442) | **0.144***** (0.0405 - 0.514) |
| Spain | 0.646 (0.265 - 1.574) | 0.560 (0.161 - 1.946) |
| Italy | 1.455 (0.664 - 3.190) | 2.655 (0.816 - 8.634) |
| France | 0.420 (0.131 - 1.346) | 0.704 (0.151 - 3.290) |
| Denmark | 0.695 (0.344 - 1.402) | 0.341 (0.112 - 1.040) |
| Greece | 1.383 (0.352 - 5.443) | 0.329 (0.0479 - 2.256) |
| Switzerland | **0.175***** (0.0546 - 0.560) | 0.344 (0.113 - 1.051) |
| Belgium | 0.767 (0.417 - 1.413) | 0.899 (0.396 - 2.039) |
| Israel | 1.350 (0.494 - 3.692) | 1.251 (0.373 - 4.190) |
| Slovenia | 1.131 (0.568 - 2.251) | 0.952 (0.363 - 2.493) |
| Estonia | **2.309***** (1.313 - 4.059) | 2.083 (0.944 - 4.597) |
|  |  |  |
| Diagnosed Diabetes X Sweden | 3.408 (0.670 - 17.33) |  |
| Diagnosed Diabetes X Spain | 2.136 (0.480 - 9.510) |  |
| Diagnosed Diabetes X Italy | 1.678 (0.448 - 6.289) |  |
| Diagnosed Diabetes X France | 2.853 (0.373 - 21.83) |  |
| Diagnosed Diabetes X Denmark | 0.450 (0.0934 - 2.172) |  |
| Diagnosed Diabetes X Greece | 1.489 (0.206 - 10.78) |  |
| Diagnosed Diabetes X Switzerland | 3.524 (0.572 - 21.72) |  |
| Diagnosed Diabetes X Belgium | 0.532 (0.139 - 2.037) |  |
| Diagnosed Diabetes X Israel | 4.264 (0.953 - 19.07) |  |
| Diagnosed Diabetes X Slovenia | 1.791 (0.434 - 7.396) |  |
| Diagnosed Diabetes X Estonia | 2.109 (0.754 - 5.900) |  |
|  |  |  |
| Diagnosed Hypertension X Sweden |  | 2.668 (0.577 - 12.33) |
| Diagnosed Hypertension X Spain |  | 1.836 (0.447 - 7.542) |
| Diagnosed Hypertension X Italy |  | 0.523 (0.137 - 2.000) |
| Diagnosed Hypertension X France |  | 0.586 (0.0872 - 3.941) |
| Diagnosed Hypertension X Denmark |  | 2.531 (0.682 - 9.399) |
| Diagnosed Hypertension X Greece |  | 6.230 (0.733 - 52.94) |
| Diagnosed Hypertension X Switzerland |  | 0.365 (0.0505 - 2.629) |
| Diagnosed Hypertension X Belgium |  | 0.556 (0.189 - 1.631) |
| Diagnosed Hypertension X Israel |  | 3.513 (0.769 - 16.05) |
| Diagnosed Hypertension X Slovenia |  | 1.616 (0.491 - 5.322) |
| Diagnosed Hypertension X Estonia |  | 1.547 (0.608 - 3.936) |
|  |  |  |
| Observations  Controls | 2,911  X | 2,911  X |
