# Supplementary material for: High unawareness of kidney dysfunction in European older adults and the importance of early detection through comorbidities
Source: PLoS One. 2025 Oct 14;20(10):e0333578. doi: 10.1371/journal.pone.0333578 (PMC12520349; doi:10.1371/journal.pone.0333578)
Supplement: S3 Table — Note: Models (1) – (4) predict the probability of CKD diagnosis among the full sample, for each age cohort (50–64), (65–74), (75–84) and (85+). Arthritis is the combined measure of rheumatoid and osteoarthritis from self-report. Country Controls are included in each model. Odds ratios presented with 95% CI in parentheses (*** p < 0.01, ** p < 0.05). (DOCX) [file pone.0333578.s003.docx]

|  | **Model (1)** | **Model (2)** | **Model (3)** | **Model (4)** |
| --- | --- | --- | --- | --- |
| VARIABLES | **P(Diag)**  **Age 50-64** | **P(Diag)**  **Age 65-74** | **P(Diag)**  **Age 75-84** | **P(Diag)**  **Age 85+** |
|  |  |  |  |  |
| Diabetes | 0.944 (0.412 - 2.160) | 1.893 (0.947 - 3.785) | 1.241 (0.598 - 2.574) | 2.818 (0.854 - 9.298) |
| Hypertension | **6.306***** (2.445 - 16.26) | 0.937 (0.470 - 1.869) | 0.881 (0.407 - 1.905) | 0.734 (0.199 - 2.701) |
| Heart Attack | 1.377 (0.530 - 3.573) | **2.112**** (1.142 - 3.906) | 1.433 (0.613 - 3.351) | 2.372 (0.609 - 9.237) |
| Stroke | 1.255 (0.288 - 5.469) | 2.607 (0.927 - 7.329) | 2.617 (0.998 - 6.864) | 1.565 (0.275 - 8.909) |
| Arthritis | 0.953 (0.464 - 1.958) | **7.039***** (3.463 - 14.31) | 2.008 (0.965 - 4.179) | 1.355 (0.486 - 3.780) |
| Cancer | **3.026**** (1.134 - 8.072) | **5.421*****(1.651 - 17.80) | **5.025***** (2.051 - 12.31) | **8.619**** (1.502 - 49.46) |
| Euro-D | 2.618** (1.081 - 6.340) | 1.418 (0.679 - 2.958) | 1.452 (0.619 - 3.404) | 2.473 (0.678 - 9.022) |
| BMI | 0.989 (0.918 - 1.065) | 0.970 (0.901 - 1.043) | 1.034 (0.937 - 1.141) | 0.999 (0.898 - 1.112) |
| Ever Smoke? | 0.894 (0.381 - 2.095) | 0.584 (0.276 - 1.236) | 1.538 (0.764 - 3.096) | 0.712 (0.141 - 3.603) |
| Physical Inactivity | 1.938 (0.755 - 4.976) | 0.943 (0.371 - 2.401) | **2.845***** (1.530 - 5.291) | 1.043 (0.303 - 3.586) |
| Alcohol in last 7 days? | 1.142 (0.412 - 3.164) | **0.435**** (0.211 - 0.897) | 0.865 (0.426 - 1.757) | 1.105 (0.326 - 3.746) |
| Ability to Make Ends Meet |  |  |  |  |
| With some difficulty | 1.205 (0.335 - 4.333) | 0.813 (0.278 - 2.377) | 1.013 (0.370 - 2.770) | 0.539 (0.108 - 2.697) |
| Fairly Easily | 1.033 (0.193 - 5.539) | 1.634 (0.514 - 5.196) | 0.889 (0.321 - 2.462) | 0.684 (0.125 - 3.732) |
| Easily | 0.685 (0.138 - 3.411) | 2.129 (0.774 - 5.850) | 1.278 (0.434 - 3.761) | 1.358 (0.316 - 5.833) |
| Education |  |  |  |  |
| Medium Educ | 0.851 (0.288 - 2.515) | **0.276***** (0.104 - 0.733) | 1.387 (0.593 - 3.244) | 0.802 (0.0459 - 14.02) |
| High Educ | 1.550 (0.271 - 8.869) | **0.309**** (0.103 - 0.920) | 1.025 (0.357 - 2.939) | 2.479 (0.451 - 13.64) |
| Female | 1.399 (0.617 - 3.170) | 1.153 (0.534 - 2.491) | 0.594 (0.257 - 1.370) | 2.130 (0.539 - 8.422) |
|  |  |  |  |  |
| Country Controls | X | X | X | X |
| Observations | 8,271 | 7,794 | 4,906 | 1,243 |
|  |  |  |  |  |
