# Supplementary material for: High unawareness of kidney dysfunction in European older adults and the importance of early detection through comorbidities
Source: PLoS One. 2025 Oct 14;20(10):e0333578. doi: 10.1371/journal.pone.0333578 (PMC12520349; doi:10.1371/journal.pone.0333578)
Supplement: S4 Table — Note: Models (1) – (4) predict the probability of CKD diagnosis among those with reported and measured CKD, for each age cohort (50–64), (65–74), (75–84) and (85+). Arthritis is the combined measure of rheumatoid and osteoarthritis from self-report. Country Controls are included in each model. Odds ratios presented with 95% CI in parentheses (*** p < 0.01, ** p < 0.05). (DOCX) [file pone.0333578.s004.docx]

|  | **(1)** | **(2)** | **(3)** | **(4)** |
| --- | --- | --- | --- | --- |
| VARIABLES | **P(Diag\|CKD)**  **Age 50-64** | **P(Diag\|CKD)**  **Age 65-74** | **P(Diag\|CKD)**  **Age 75-84** | **P(Diag\|CKD)**  **Age 85+** |
|  |  |  |  |  |
| Diabetes | **0.0773***** (0.017 - 0.36) | 0.809 (0.348 - 1.878) | 1.341 (0.619 - 2.906) | 2.673 (0.672 - 10.62) |
| Hypertension | 1.850 (0.522 - 6.561) | 0.688 (0.310 - 1.528) | 0.658 (0.282 - 1.533) | 0.581 (0.146 - 2.310) |
| Heart Attack | 0.488 (0.0910 - 2.618) | 1.276 (0.517 - 3.150) | 1.236 (0.536 - 2.853) | 2.026 (0.520 - 7.895) |
| Stroke | 1.744 (0.210 - 14.47) | 1.718 (0.444 - 6.645) | 1.414 (0.530 - 3.777) | 1.652 (0.226 - 12.09) |
| Arthritis | 4.389 (0.949 - 20.30) | **7.543***** (3.265 - 17.43) | 1.870 (0.823 - 4.250) | 1.900 (0.629 - 5.735) |
| Cancer | 0.939 (0.0655 - 13.46) | **7.365***** (2.252 - 24.08) | **7.382***** (2.412 - 22.59) | 6.001 (0.873 - 41.24) |
| Euro-D | 1.312 (0.314 - 5.474) | 2.001 (0.860 - 4.655) | 1.074 (0.490 - 2.350) | 2.501 (0.493 - 12.69) |
| BMI | 0.926 (0.827 - 1.036) | 0.938 (0.876 - 1.005) | 1.006 (0.920 - 1.099) | 0.974 (0.867 - 1.096) |
| Ever Smoke? | 0.640 (0.213 - 1.918) | 0.373 (0.139 - 1.000) | 1.027 (0.445 - 2.373) | 1.082 (0.198 - 5.909) |
| Physical Inactivity | 2.824 (0.546 - 14.61) | 1.059 (0.376 - 2.983) | **2.449**** (1.175 - 5.107) | 0.563 (0.128 - 2.481) |
| Alcohol in last 7 days? | 1.566 (0.418 - 5.872) | 0.558 (0.250 - 1.243) | 1.109 (0.518 - 2.375) | 0.831 (0.256 - 2.699) |
| Ability to Make Ends Meet |  |  |  |  |
| With some difficulty | 0.764 (0.127 - 4.586) | 1.003 (0.280 - 3.599) | 0.975 (0.321 - 2.966) | 0.860 (0.159 - 4.639) |
| Fairly Easily | 0.748 (0.123 - 4.560) | 2.385 (0.735 - 7.741) | 0.677 (0.197 - 2.329) | 0.982 (0.136 - 7.099) |
| Easily | 1.379 (0.230 - 8.279) | **5.135**** (1.445 - 18.25) | 1.175 (0.342 - 4.028) | 1.579 (0.266 - 9.378) |
| Education |  |  |  |  |
| Medium Educ | 0.805 (0.138 - 4.708) | 0.483 (0.172 - 1.357) | 1.463 (0.554 - 3.863) | 0.598 (0.0776 - 4.603) |
| High Educ | 0.652 (0.112 - 3.800) | 0.364 (0.0802 - 1.656) | 1.117 (0.322 - 3.874) | 2.093 (0.364 - 12.04) |
| Female | 0.270 (0.0519 - 1.399) | 0.414 (0.135 - 1.272) | 0.407** (0.173 - 0.957) | 1.135 (0.277 - 4.648) |
|  |  |  |  |  |
| Country Controls | X | X | X | X |
| Observations | 210 | 673 | 1,341 | 671 |
