# Supplementary material for: High unawareness of kidney dysfunction in European older adults and the importance of early detection through comorbidities
Source: PLoS One. 2025 Oct 14;20(10):e0333578. doi: 10.1371/journal.pone.0333578 (PMC12520349; doi:10.1371/journal.pone.0333578)
Supplement: S5 Table — Note: Models (1) – (4) predict the probability of CKD diagnosis among those with eGFRcys levels below 60 mL/min/1.73 m2, for each age cohort (50–64), (65–74), (75–84) and (85+). Country Controls are included in each model. Odds ratios presented with 95% CI in parentheses (*** p < 0.01, ** p < 0.05). (DOCX) [file pone.0333578.s005.docx]

|  | **(1)** | **(2)** | **(3)** | **(4)** |
| --- | --- | --- | --- | --- |
| VARIABLES | **P(Diag \| GFR<60)**  **Age 50-64** | **P(Diag \| GFR<60)**  **Age 65-74** | **P(Diag \| GFR<60)**  **Age 75-84** | **P(Diag \| GFR<60)**  **Age 85+** |
|  |  |  |  |  |
| Diabetes | 0.0102 (1.49e-05 - 6.926) | 3.429 (0.710 - 16.57) | 1.080 (0.421 - 2.771) | **15.06***** (3.151 - 71.94) |
| Hypertension | **96.80***** (3.703 - 2,531) | 1.318 (0.344 - 5.049) | 0.675 (0.257 - 1.771) | **0.0840***** (0.0191 - 0.369) |
| Heart Attack | 0.430 (0.00981 - 18.81) | 2.820 (0.785 - 10.12) | 1.969 (0.751 - 5.159) | **9.970***** (2.749 - 36.16) |
| Stroke | 131.2 (0.326 - 52,877) | 1.329 (0.186 - 9.485) | 1.988 (0.678 - 5.831) | 0.651 (0.0271 - 15.67) |
| Arthritis | 2.110 (0.0683 - 65.17) | **17.83***** (4.581 - 69.41) | 1.111 (0.404 - 3.055) | 0.708 (0.161 - 3.104) |
| Cancer | 1.000 (0.0648 - 15.42) | **14.16***** (3.132 - 64.00) | **13.55***** (3.814 - 48.14) | **11.84**** (1.322 - 106.0) |
| Euro-D | 0.274 (0.0185 - 4.052) | 3.257 (0.550 - 19.30) | 0.884 (0.346 - 2.257) | 1.891 (0.296 - 12.09) |
| BMI | 1.077 (0.845 - 1.373) | 0.921 (0.842 - 1.007) | 1.011 (0.901 - 1.135) | 0.913 (0.770 - 1.081) |
| Ever Smoke? | **0.0206****(0.001 - 0.424) | 0.691 (0.148 - 3.224) | 0.845 (0.353 - 2.024) | 1.808 (0.345 - 9.465) |
| Physical Inactivity | 42.83 (0.776 - 2,365) | 1.464 (0.242 - 8.847) | 2.240 (0.887 - 5.655) | 1.495 (0.201 - 11.11) |
| Alcohol in last 7 days? | **0.0253***** (0.004 - 0.170) | 1.042 (0.221 - 4.912) | 1.228 (0.488 - 3.090) | 2.376 (0.634 - 8.914) |
| Ability to Make Ends Meet |  |  |  |  |
| With some difficulty | 0.686 (0.0160 - 29.45) | 0.477 (0.0575 - 3.949) | 0.904 (0.240 - 3.401) | 0.673 (0.0355 - 12.77) |
| Fairly Easily | 7.312 (0.225 - 237.7) | **0.0621**** (0.00545 - 0.707) | 0.451 (0.115 - 1.772) | 3.816 (0.0355 - 12.77) |
| Easily | 14.04 (0.525 - 375.6) | 3.704 (0.486 - 28.25) | 0.434 (0.104 - 1.807) | 1.486 (0.137 - 16.18) |
| Education |  |  |  |  |
| Medium Educ | 10.06 (0.592 - 170.9) | 0.319 (0.0658 - 1.550) | 2.613 (0.960 - 7.113) | 0.403 (0.0901 - 1.798) |
| High Educ | 58.35 (0.867 - 3,927) | 0.226 (0.0371 - 1.375) | 2.718 (0.722 - 10.23) | **9.775**** (1.673 - 57.13) |
| Female | 1.115 (0.130 - 9.573) | 0.630 (0.137 - 2.900) | 0.460 (0.177 - 1.195) | 1.939 (0.426 - 8.829) |
|  |  |  |  |  |
| Country Controls | X | X | X | X |
| Observations | 131 | 555 | 1,264 | 659 |
