# Supplementary material for: High unawareness of kidney dysfunction in European older adults and the importance of early detection through comorbidities
Source: PLoS One. 2025 Oct 14;20(10):e0333578. doi: 10.1371/journal.pone.0333578 (PMC12520349; doi:10.1371/journal.pone.0333578)
Supplement: S6 Table — Note: Models (1) predicts the probability of CKD diagnosis among the full sample. Model (2) predicts the probability of CKD diagnosis among those with reported and measured CKD. Models (3) predicts probability of CKD diagnosis among those with reported and measured CKD, with eGFRcys levels below 60 mL/min/1.73 m2. Each model includes the categorical by categorical interaction of the number of comorbidities with age groups (50–64), (65–74), (75–84) and (85+), with no comorbidities and age (50–64) as comparison groups. Country and demographic controls are included in each model, replicating Table 3 in the main text. Odds ratios presented with 95% CI in parentheses (*** p < 0.01, ** p < 0.05). (DOCX) [file pone.0333578.s006.docx]

|  | **Model (1)** | **Model (2)** | **Model (3)** |
| --- | --- | --- | --- |
| VARIABLES | **P(Diag)** | **P(Diag\|CKD)** | **P(Diag \| GFR<60)** |
| Number of Comorbities |  |  |  |
| One | **3.561**** (1.090 - 11.63) | 2.184 (0.448 - 10.63) | **30.26***** (2.288 - 400.3) |
| Two | **4.430**** (1.294 - 15.17) | 2.088 (0.386 - 11.30) | **25.27**** (1.631 - 391.7) |
| Three | **5.555**** (1.384 - 22.29) | 1.696 (0.232 - 12.39) | **28.48**** (1.283 - 632.3) |
| Four or more | **27.80***** (5.334 - 144.9) | 4.982 (0.659 - 37.66) | **40.90**** (1.626 - 1,029) |
| Age Group |  |  |  |
| Age 65-74 | 1.298 (0.350 - 4.819) | 0.276 (0.0474 - 1.607) | 0.742 (0.0457 - 12.04) |
| Age 75-84 | 1.158 (0.242 - 5.554) | **0.0659***** (0.01 - 0.437) | 1.906 (0.112 - 32.32) |
| Age 85+ | 4.661 (0.506 - 42.93) | **0.0859**** (0.008 - 0.916) | 0.153 (0.00602 - 3.905) |
| Interaction of Comorbidities and Age Group |  |  |  |
| One X Age 65-74 | 0.805 (0.149 - 4.363) | 1.089 (0.135 - 8.788) | 0.213 (0.0083 - 5.469) |
| One X Age 75-84 | 1.535 (0.252 - 9.340) | 1.661 (0.208 - 13.28) | 0.0747 (0.0038 - 1.479) |
| One X Age 85+ | 0.239 (0.0163 - 3.512) | 0.430 (0.0248 - 7.469) | 0.755 (0.0219 - 26.02) |
| Two X Age 65-74 | 0.825 (0.164 - 4.161) | 0.710 (0.0895 - 5.637) | 0.125 (0.00460 - 3.404) |
| Two X Age 75-84 | 1.077 (0.164 - 7.070) | 1.174 (0.119 - 11.53) | 0.0930 (0.00377 - 2.292) |
| Two X Age 85+ | 0.271 (0.0200 - 3.661) | 0.475 (0.0287 - 7.876) | 0.120 (0.00355 - 4.061) |
| Three X Age 65-74 | 1.297 (0.216 - 7.774) | 1.035 (0.0946 - 11.32) | 0.578 (0.0169 - 19.79) |
| Three X Age 75-84 | 2.566 (0.360 - 18.30) | 4.488 (0.395 - 51.01) | 0.155 (0.00499 - 4.811) |
| Three X Age 85+ | 1.075 (0.0865 - 13.37) | 3.501 (0.202 - 60.74) | 3.104 (0.0741 - 130.1) |
| Four or more X Age 65-74 | 1.276 (0.155 - 10.50) | 2.238 (0.168 - 29.76) | 2.849 (0.0628 - 129.3) |
| Four or more X Age 75-84 | 0.313 (0.0338 - 2.906) | 0.807 (0.0604 - 10.78) | 0.113 (0.00279 - 4.615) |
| Four or more X Age 85+ | 0.155 (0.00729 - 3.309) | 0.926 (0.0327 - 26.24) | 1.961 (0.0256 - 150.3) |
|  |  |  |  |
| Observations  Demographic Controls  Country Controls | 22,386 X  X | 2,911  X X | 2,650  X X |
