# Supplementary material for: High unawareness of kidney dysfunction in European older adults and the importance of early detection through comorbidities
Source: PLoS One. 2025 Oct 14;20(10):e0333578. doi: 10.1371/journal.pone.0333578 (PMC12520349; doi:10.1371/journal.pone.0333578)
Supplement: S7 Table — Note: Models (1) predicts the probability of CKD diagnosis among the full sample. Model (2) predicts the probability of CKD diagnosis among those with reported and measured CKD. Models (3) predicts probability of CKD diagnosis among those with reported and measured CKD, with eGFRcys levels below 60 mL/min/1.73 m2. Each model includes the triple interaction of the gender, age group (50–64), (65–74), (75–84) and (85+), and education (low educ = ISCED 1997 cat 0–2), (medium educ = ISCED 1997 cat 3,4), (high educ = ISCED 1997 cat 5,6). Male, age group (50–64), and low education are the baseline categories. Health, demographic, and country controls are included in each model, replicating Table 3 in the main text. Odds ratios presented with 95% CI in parentheses (*** p < 0.01, ** p < 0.05). (DOCX) [file pone.0333578.s007.docx]

|  | **Model (1)** | **Model (2)** | **Model (3)** |
| --- | --- | --- | --- |
| VARIABLES | **P(Diag)** | **P(Diag\|CKD)** | **P(Diag \| GFR<60)** |
|  |  |  |  |
| Age 65-74 | 1.260 (0.328 - 4.839) | **0.102**** (0.0171 - 0.608) | **0.0977**** (0.0120 - 0.799) |
| Age 75-84 | 1.525 (0.503 - 4.627) | **0.0510***** (0.010 - 0.256) | **0.0399***** (0.00549 - 0.290) |
| Age 85+ | 0.846 (0.159 - 4.510) | **0.0128***** (0.0014 - 0.120) | **0.0219***** (0.00190 - 0.252) |
|  |  |  |  |
| Medium Educ | 0.588 (0.149 - 2.326) | 1.319 (0.177 - 9.845) | **0.0662**** (0.00517 - 0.847) |
| High Educ | 0.719 (0.143 - 3.623) | 0.288 (0.0375 - 2.209) | 0.0755 (0.00438 - 1.299) |
|  |  |  |  |
| Age 65-74 X Medium Educ | 0.687 (0.113 - 4.168) | 0.549 (0.0493 - 6.126) | 3.903 (0.164 - 92.97) |
| Age 65-74 X High Educ | 0.804 (0.0976 - 6.617) | 5.623 (0.418 - 75.58) | 3.396 (0.107 - 108.3) |
| Age 75-84 X Medium Educ | 2.961 (0.524 - 16.72) | 1.505 (0.153 - 14.82) | **31.50**** (1.794 - 553.1) |
| Age 75-84 X High Educ | 2.193 (0.295 - 16.30) | 6.541 (0.535 - 79.96) | **45.93**** (1.703 - 1,239) |
| Age 85+ X Medium Educ | 4.762 (0.339 - 66.98) | 1.869 (0.0738 - 47.37) | 8.138 (0.261 - 253.3) |
| Age 85+ X High Educ | 3.575 (0.243 - 52.63) | 10.29 (0.474 - 223.4) | **52.78**** (1.378 - 2,021) |
|  |  |  |  |
| Female | 0.754 (0.168 - 3.381) | 0.259 (0.0294 - 2.282) | 0.214 (0.0113 - 4.085) |
|  |  |  |  |
| Age 65-74 X Female | 1.866 (0.282 - 12.36) | 3.925 (0.304 - 50.72) | 1.579 (0.0506 - 49.25) |
| Age 75-84 X Female | 1.051 (0.197 - 5.598) | 2.157 (0.210 - 22.11) | 2.684 (0.119 - 60.58) |
| Age 85+ X Female | 3.986 (0.470 - 33.81) | 10.57 (0.631 - 177.2) | 6.072 (0.185 - 199.4) |
|  |  |  |  |
| Medium Educ X Female | 1.762 (0.277 - 11.21) | 1.627 (0.105 - 25.09) | 14.79 (0.336 - 652.1) |
| High Educ X Female | 3.037 (0.310 - 29.74) | 4.561 (0.266 - 78.25) | 16.45 (0.348 - 777.6) |
|  |  |  |  |
| Age 65-74 X Medium Educ X Female | 0.650 (0.0553 - 7.641) | 0.608 (0.0212 - 17.48) | 0.351 (0.00250 - 49.38) |
| Age 65-74 X High Educ X Female | 0.238 (0.0117 - 4.844) | 0.0390 (0.00106 - 1.442) | 0.250 (0.00192 - 32.46) |
| Age 75-84 X Medium Educ X Female | 0.253 (0.0234 - 2.733) | 0.236 (0.00992 - 5.601) | 0.0551 (0.000746 - 4.072) |
| Age 75-84 X High Educ X Female | **0.0239***** (0.00157 - 0.364) | **0.0216**** (0.000768 - 0.606) | **0.00174***** (2.11e-05 - 0.144) |
| Age 85+ X Medium Educ X Female | **0.00474***** (0.0002 - 0.152) | **0.00544**** (8.89e-05 - 0.334) | **0.00513**** (3.90e-05 - 0.673) |
| Age 85+ X High Educ X Female | **0.0272**** (0.00084 - 0.885) | **0.0122**** (0.000234 - 0.637) | **0.00652**** (5.24e-05 - 0.810) |
| Health controls  Demographic controls | X  X | X X | X X |
| Observations | 22,386 | 2,911 | 2,650 |
