# Supplementary material for: High unawareness of kidney dysfunction in European older adults and the importance of early detection through comorbidities
Source: PLoS One. 2025 Oct 14;20(10):e0333578. doi: 10.1371/journal.pone.0333578 (PMC12520349; doi:10.1371/journal.pone.0333578)
Supplement: S8 Table — Note: Models (1) predicts the probability of CKD diagnosis among the full sample. Model (2) predicts the probability of CKD diagnosis among those with reported and measured CKD. Models (3) predicts probability of CKD diagnosis among those with reported and measured CKD, with eGFRcys levels below 60 mL/min/1.73 m2. We present the average marginal effects of Table 8, held at each combination of age group, gender, and education. The margins come from Table 8, which includes the triple interaction of the gender, age group (50–64), (65–74), (75–84) and (85+), and education (low educ = ISCED 1997 cat 0–2), (medium educ = ISCED 1997 cat 3,4), (high educ = ISCED 1997 cat 5,6). Male, age group (50–64), and low education are the baseline categories. Health, demographic, and country controls are included in each model, replicating Table 3 in the main text. (*** p < 0.01, ** p < 0.05). (DOCX) [file pone.0333578.s008.docx]

|  | **Model (1)** | **Model (2)** | **Model (3)** |
| --- | --- | --- | --- |
| VARIABLES | **P(Diag)** | **P(Diag\|CKD)** | **P(Diag \| GFR<60)** |
|  |  |  |  |
| 50-64, Low Educ, Male | **0.0164**** (0.00802) | **0.680***** (0.138) | **0.540***** (0.180) |
| 50-64, Low Educ,Female | 0.0125 (0.00720) | **0.410**** (0.159) | 0.265 (0.179) |
| 50-64, Medium Educ, Male | **0.00987**** (0.00482) | **0.729***** (0.119) | 0.124 (0.0873) |
| 50-64, Medium Educ, Female | **0.0130***** (0.00391) | **0.566***** (0.102) | **0.262**** (0.113) |
| 50-64, High Educ, Male | 0.0120 (0.00730) | **0.431***** (0.139) | 0.136 (0.106) |
| 50-64, High Educ, Female | 0.0261 (0.0154) | **0.465***** (0.144) | **0.299**** (0.130) |
|  |  |  |  |
| 65-74, Low Educ, Male | 0.0203 (0.0106) | **0.241***** (0.0867) | **0.163**** (0.0647) |
| 65-74, Low Educ, Female | **0.0279***** (0.00869) | **0.244***** (0.0581) | **0.0734**** (0.0359) |
| 65-74, Medium, Male | **0.00858***** (0.00320) | **0.195***** (0.0677) | 0.0590 (0.0391) |
| 65-74, Medium Educ, Female | **0.0136**** (0.00532) | **0.196***** (0.0628) | 0.0924 (0.0631) |
| 65-74, High Educ, Male | 0.0121 (0.00681) | **0.323***** (0.123) | 0.0586 (0.0490) |
| 65-74, High Educ, Female | 0.0123 (0.00733) | **0.0984****(0.0417) | 0.0765 (0.0506) |
|  |  |  |  |
| 75-84, Low Educ, Male | **0.0243***** (0.00742) | **0.149***** (0.0420) | **0.0851***** (0.0293) |
| 75-84, Low Educ, Female | **0.0196***** (0.00635) | **0.0947***** (0.0257) | **0.0546**** (0.0217) |
| 75-84, Medium Educ, Male | **0.0401**** (0.0177) | **0.240***** (0.0766) | **0.146**** (0.0628) |
| 75-84, Medium Educ, Female | **0.0154**** (0.00654) | **0.0755**** (0.0311) | **0.0836**** (0.0390) |
| 75-84, High Educ, Male | **0.0368**** (0.0182) | **0.232**** (0.0972) | **0.204**** (0.0893) |
| 75-84, High Educ, Female | **0.00240**** (0.00109) | **0.0212**** (0.0101) | 0.00664 (0.00534) |
|  |  |  |  |
| 85+, Low Educ, Male | 0.0140 (0.00933) | 0.0477 (0.0365) | 0.0524 (0.0361) |
| 85+, Low Educ, Female | **0.0387***** (0.0128) | **0.112***** (0.0312) | **0.0651***** (0.0232) |
| 85+, Medium Educ, Male | 0.0363 (0.0309) | 0.103 (0.0848) | 0.0308 (0.0249) |
| 85+, Medium Educ, Female | 0.00104 (0.000906) | 0.00322 (0.00252) | 0.00345 (0.00325) |
| 85+, High Educ, Male | 0.0336 (0.0271) | 0.119 (0.0725) | 0.151 (0.0835) |
| 85+, High Educ, Female | 0.00911 (0.00607) | 0.0230 (0.0159) | 0.0316 (0.0244) |
| Health controls  Demographic controls | X  X | X X | X X |
| Observations | 22,386 | 2,911 | 2,650 |
