# Supplementary material for: High unawareness of kidney dysfunction in European older adults and the importance of early detection through comorbidities
Source: PLoS One. 2025 Oct 14;20(10):e0333578. doi: 10.1371/journal.pone.0333578 (PMC12520349; doi:10.1371/journal.pone.0333578)
Supplement: S9 Table — Note: Odds ratios presented with 95% in parentheses (*** p < 0.01, ** p < 0.05). Undiagnosed Diabetes is defined by having an Hba1c level above 6.5 mg/dL and not reporting a doctor’ diagnosis of diabetes/not taking any medication for diabetes. Model (1) predicts the probability of CKD diagnosis among the full sample. Models (2) predicts the probability of CKD diagnosis among those with reported and measured CKD. Models (3) predicts the probability of CKD diagnosis among those with reported and measured CKD, with eGFRcys levels below 60 mL/min/1.73 m2. Health controls include: hypertension, heart attack, stroke, arthritis, depression (euro-d), bmi, physical inactivity, and alcohol consumption in the last 7 days. Demographic controls include: ability to make ends meet, education, age, gender, and country dummies. (DOCX) [file pone.0333578.s009.docx]

|  | **Model (1)** | **Model (2)** | **Model (3)** |
| --- | --- | --- | --- |
| VARIABLES | **P(Diag)** | **P(Diag\|CKD)** | **P(Diag \| GFR<60)** |
|  |  |  |  |
| 1.Diagnosed Diabetes | 1.275 (0.736 - 2.211) | 0.932 (0.469 - 1.854) | 1.136 (0.459 - 2.815) |
|  |  |  |  |
| 2.Undiagnosed Diabetes | 1.066 (0.474 - 2.397) | 1.158 (0.488 - 2.743) | 0.282 (0.0743 - 1.072) |
|  |  |  |  |
| Health controls  Demographic controls | X  X | X X | X X |
| Observations | 18,599 | 2,406 | 2,186 |
